# Occurrence and Genetic Correlations of *Yersinia* spp. Isolated from Commensal Rodents in Northeastern Poland

**DOI:** 10.3390/pathogens10101247

**Published:** 2021-09-27

**Authors:** Aleksandra Platt-Samoraj, Klaudia Kończyk-Kmiecik, Tadeusz Bakuła

**Affiliations:** 1 Department of Epizootiology, Faculty of Veterinary Medicine, University of Warmia and Mazury in Olsztyn, Oczapowskiego 13 Str., 10-718 Olsztyn, Poland; klaudia.konczyk@uwm.edu.pl; 2 Department of Veterinary Prevention and Feed Hygiene, Faculty of Veterinary Medicine, University of Warmia and Mazury in Olsztyn, Oczapowskiego 13 Str., 10-718 Olsztyn, Poland; bakta@uwm.edu.pl

**Keywords:** *Yersinia enterocolitica*, *Yersinia pseudotuberculosis*, *Yersinia kristensenii*, *ail*, *ystB*, *inv*, rodents, vectors

## Abstract

Rodents can be a potential *Yersinia* spp. vector responsible for farm facilities contamination. The aim of the study was to determine the prevalence of *Yersinia* spp. in commensal rodents found in the farms and fodder factory areas to characterize the obtained isolates and epidemiological risk. Intestinal samples were subjected to bacteriological, bioserotype, and PCR examination for virulence markers *ail, ystA, ystB,* and *inv* presence. *Yersinia* spp. was isolated from 43 out of 244 (17.6%) rodents (*Apodemus agrarius* n = 132, *Mus musculus* n = 102, *Apodemus sylvaticus* n = 8, *Rattus norvegicus* n = 2). *Y. enterocolitica* was isolated from 41 rodents (16.8%), and from one *Y. pseudotuberculosis* and one *Y. kristensenii*. In three cases, two *Y. enterocolitica* isolates were obtained from one rodent. All *Y. enetrocolitica* contained *ystB* and belonged to biotype 1A, considered as potentially pathogenic. One isolate additionally had the *ail* gene typical for pathogenic strains. The sequence analysis of the *ystB, ail,* and *inv* fragments showed a high similarity to those from clinical cases. The current study revealed a high prevalence of *Y. enetrocolitica* among commensal rodents, but the classification of all of *Y. enterocolitica* isolates into biotype 1A and the sporadic isolation of * Y. pseudotuberculosis* do not indicate a high epidemiological risk.

## 1. Introduction

The genus *Yersinia* belongs to the *Enterobacteriaceae* family and includes at least 19 species, the list of which is still being revised with newly discovered species [1]. Yersiniosis is a zoonotic gastrointestinal disease caused by two enteropathogenic *Yersinia* (*Y*.) species, i.e., *Y. enterocolitica* and *Y. pseudotuberculosis* [2]. Both pathogens are widely disseminated in the environment. The epidemiology and mechanisms of the circulation of the microorganism are complex and not fully understood. *Yersinia* spp. can be isolated from animals and food, as well as from water, plants, and soil contaminated by feces of infected animals [3].

According to a recent EFSA (European Food Safety Authority) zoonoses report, yersiniosis was the fourth most frequently reported zoonosis in humans in 2019. There were 6961 confirmed cases in Europe, 648 of which required hospitalization [4]. The disease is usually associated with diarrhea as acute gastroenteritis or pseudoappendicitis, but it can also cause long-term extraintestinal sequels such as erythema nodosum or reactive arthritis. Yersiniosis can also lead to sepsis, which is often fatal [5].

Based on biochemical properties, such as esculin, xylose, and trehalose fermentation, and the production of pyrazinamidase and tween esterase, six biotypes (BT) of *Y. enterocolitica* were distinguished: 1A, 1B, and 2, 3, 4, and 5. Depending on the lipopolysaccharide cell wall diversity, over 60 serotypes of *Y. enterocolitica* were distinguished [6,7,8]. Microorganisms belonging to BT 1B, 2–5 are mainly isolated from clinical cases. *Y. enterocolitica* belonging to BT 1A until recently were considered non-pathogenic because they lack the pathogenicity plasmid pYV (Yersinia virulence plasmid), which contributes to survival and multiplication of bacteria in host tissues, the chromosomal *ail* and *inv* genes which encode products responsible for the invasion and adhesion processes to the intestinal epithelial cells, as well as the *ystA* encoding enterotoxin YstA [5,9]. However, recent studies have increasingly drawn attention to the potential pathogenic properties of certain strains of the BT 1A which can carry the *ystB* gene responsible for the production of YstB enterotoxin [10]. Compared to other *Y. enterocolitica* biotypes, BT1A is the most heterogeneous and contains the most serotypes [7,11]. In the case of *Y. pseudotuberculosis*, all strains isolated from clinical cases are considered pathogenic [12]. 

For the detection of Yersinia, in addition to traditional bacteriological methods, PCR is commonly used to search for virulence markers, both plasmid, such as *myfA, yadA,* and chromosomal, such as *inv, ail, yst*, encoding the production of proteins responsible for the penetration and colonization of the host organism and resistance to the immune mechanisms of the infected organism [9,13].

*Yersinia* species of clinical importance include the previously mentioned *Y. enterocolitica* and *Y. pseudotuberculosis* as the factors of yersiniosis and *Y. pestis*, which is a plague factor. The remaining *Yersinia* species, apart from the fish pathogenic *Y. ruckeri*, are generally considered to be non-pathogenic, conditionally pathogenic or of unknown pathogenetic potential [3]. Many species of livestock, companion animals and free-living animals are susceptible to infection with *Yersinia* spp. The main reservoir of *Y. enterocolitica* is considered to be pigs, which often have an asymptomatic carrier and shed the microorganism [14]. Pork meat and food products contaminated with the microorganism excreted by this animal species are considered the most common causes of yersiniosis in humans. The environment in which pigs live is often contaminated with this microorganism, and the elimination of *Y. enterocolitica* from piggeries and their surroundings is hampered by the existence of numerous vectors. One of the most important vectors of the factors are rodents, which can transmit pathogens to domestic animals and to humans [15]. It has been shown that pigs and wild rodents are the reservoirs of the most dangerous high-pathogenic bioserotype 1B/O: 8 *Y. enterocolitica* in Japan [16].

Compared to *Y. enterocolitica*, much less is known about the transmission pathways and reservoirs of *Y. pseudotuberculosis*, which is less often isolated from livestock and humans but is responsible for the most severe clinical cases of yersiniosis [12].

Commensal rodents are a reservoir for many infectious diseases, but they more often act as a vector. They live in various environments to which they have adapted their body structure and way of life. However, in winter, they inhibit households and livestock facilities. If they are carriers of zoonotic agents, they become a threat to the health of humans, companion animals and farm animals and significantly hinder the control of diseases caused by these pathogens [17]. 

Recent studies have revealed that rodents have developed unique disease tolerance mechanisms that do not impair their reproductive capacity. It is predicted that they may act as super reservoirs of zoonoses in the future, mainly due to the fact that they can carry several zoonotic pathogens simultaneously. They quickly reach sexual maturity, pregnancies are short, and their offspring are numerous. Additionally, they can spread pathogens over long distances. Rodents that host pathogens may be responsible for transferring them to other buildings within the farm, to neighboring farms and contaminating the environment around them [18]. Feed, which is contaminated at the place of production or storage, is essential in introducing pathogens into herds. In this case, the main blame is also placed on rodents, which, despite biosecurity barriers, often manage to penetrate sites that are rich in food [19].

To date, no studies have been conducted in Poland that could even roughly estimate the importance of commensal rodents which periodically inhabit farm buildings as a vector of pathogenic yersinia and, thus, to what extent they contribute to the occurrence of yersiniosis in humans. The authors’ previous research confirmed the circulation of *Y. enterocolitica* among wild animals such as beavers and game animals and among small wild forest and field rodents. A high genetic relationship was also demonstrated between yersinia isolated from these animal species [20,21,22]. 

The aim of the present research is to determine the prevalence of *Yersinia* spp.in commensal rodents found in the area of farm facilities and fodder factories and to characterize and analyze the genetic correlations of the obtained isolates to assess the associated public health risk.

## 2. Material and Methods

The study was carried out on 244 rodents obtained from areas belonging to farm buildings and fodder factories in northeastern Poland. The examined facilities included ten locations: four piggeries, two barns and one poultry farm, two fodder factories and an animal house of the Department of Epizootiology, Faculty of Veterinary Medicine, University of Warmia and Mazury in Olsztyn, Poland. The study included 132 striped field mice (*Apodemus agrarius*), 102 house mice (*Mus musculus domesticus*), eight field mice (*Apodemus sylvaticus*), and two brown rats (*Rattus norvegicus*). In the vicinity of the fodder factories, 107 rodents were collected, 63 originated vicinity of from pig farms, 29 from a vicinity of poultry farm, 43 from vicinity of cattle farms, and 11 rodents were caught in the university animal building.

The animals were caught during deratization campaigns as part of the protection program against pests. Ethical approval was not required because the animals were not sacrificed for research purposes. Rodent control was carried out using Rodenticide bait stations and a snap trap in one facility. The rodents came from two successive fall campaigns from September to December in 2019 and 2020.

Immediately after the animals were delivered to the laboratory, the small intestines were collected, and the rats’ livers, kidneys, lungs, line lymph nodes, and spleens were also collected. The samples were crushed and vortexed. Following this, 200 μL of each sample suspension was placed in 10 mL of PSB medium (a peptone sorbitol and bile salts medium prepared according to PN-EN ISO 10273) and incubated at 4 ℃ for 21 days. After this time, the culture was transferred by 10 μL loop onto CIN agar (Yersinia selective Agar with Yersinia selective agar supplement, MerckKgaA, Germany) in duplicate, with and without alkali treatment. From each tube, 0.5 mL of culture was placed for 20 s in 4.5 mL of 0.5% KOH in 0.5% NaCl, and a loopful of the sample was transferred to the CIN agar and, at the same time, a second loopful was transferred directly from the PBS medium to the agar medium, which was then incubated at 30 ℃ for 48 h. For biochemical identification, 1 to 5 typical colonies (a pink to red center surrounded by a transparent border) from each culture were subjected to procedures in accordance with the PN-EN ISO 10273 standard for the initial selection. 

BT of *Y. enterocolitica* was identified according to the method proposed by Wauters [6] and described in the PN-EN ISO 10273 standard [23]. The isolates were tested for the presence of salicin acid production, esculin hydrolysis, xylose acid production, pyrazinamidase activity, and nitrate reduction. The API 20E test (bioMerieux, Marcy-l’Étoile, France) was used to determine the BT of *Y. pseudotuberculosis* by incubating the test strip at 25 °C instead of 37 °C for 20–24 h [6,24].

Serotypic affiliation was determined on the basis of slide agglutination test results using commercial diagnostic sera O:3, O:5, O:27, O:8, and O:9 (Sifin, Berlin, Germany) according to the manufacturers’ guidance. Bacterial cells were obtained from a 24-h blood agar culture (Merc, Berlin, Germany) of examined isolates. Isolates that did not react with any of the sera were designated NI (non-identified).

The course of Multiplex PCR related to the amplification of *Y. enterocolitica ail, ystA,* and *ystB* gene fragments was carried out with primers specified in Table 1. The primers were synthesized in the DNA Sequencing Laboratory of the Polish Academy of Sciences, Oligo, Warsaw, Poland. The reaction was performed using a HotStartTagPlus DNA Polimerase (Qiagen) and a HotStart Master Kit (QIagen). The following PCR protocol was adopted: final concentration of MgCl_2_—1.5 nM, initial denaturation at 95 ℃ for 300 s, followed by 30 cycles of DNA amplification: denaturation at 94 ℃ for 45 s, annealing at 54 ℃ for 30 s, polymerization at 72 ℃ for 60 s, and final polymerization at 72 ℃ for 10 min. The reaction was performed in a Mastercycler (Eppendorf, Hamburg, Germany). The products were separated by electrophoresis in 2% agarose gel with the Midori Green Advanced DNA strain (Nippon Genetics Europe GmbH, Düren, Germany) in 1x TAE buffer. The PCR results were analyzed and archived using the GelDoc gel documentation system (Quantity One analysis software, Bio-Rad, Milan, Italy). The specificity of the reaction was confirmed by sequencing the obtained amplicons.

Nucleotides were sequenced with longer *ystB* primers (263bp) [YSTBF—5′GGA CAC CGC ACA GCT TAT ATT TT 3′, YSTBR—5′ GCA CAG GCA GGA TTG CAA CA 3′], while for *inv* sequencing, new primers were developed, using the Prime Blast program, for longer amplicons (567 bp): INVF 1: -5 ′GGCAGATCCTATTCCAGATG-3′, INVR 2: 5′CTCACCGAATAACTTGGGAA-3′). The amplicons were cleaned with the Klin-up Purification Kit (A&A Biotechnology, Gdańsk, Poland) according to the manufacturer’s recommendations. Purified amplicons were directly sequenced in both directions (Genomed S.A., Poland). Multiple sequence alignment was carried out in CLUSTAL W [27]. Nucleotide and amino acid sequences were identified using BIOEDIT v.7.2.0 software. The nucleotide sequences of *ystB, ail* and *inv* analyzed in this study are available in the GeneBank [MZ496229–MZ496272] and [MZ491080–MZ491082].

## 3. Results

*Yersinia* spp. was isolated from intestinal samples from 17.6% (n = 43/244) of the tested animals, including *Y. enterocolitica* from 16.8% of the rodents (n = 41/244) and *Y. pseudotuberculosis* from one *M. musculus* and *Y. kristensenii* also from one *M. musculus*. In three cases, two different *Y. enterocolitica* isolates were obtained from two *A. agrarius* and one *R. norvegicust*. In total, 46 isolates were subjected to further studies. The results of the bacteriological study for the presence of *Yersinia* spp. confirmed by PCR are presented in Table 2. 

Of the 102 examined *M. musculus*, 18 were *Y. enterocolitica*-positive (17.6%, n = 18/102). A slightly lower percentage of infections was found among *A. agrarius*, in which, out of 132 examined mice, *Y. enterocolitica* was found in 21 mice (15.9%, n = 132/21). *Y. enterocolitica* was isolated from intestinal samples of both examined rats. The bacteria were also cultured from rat internal organs, i.e., the liver, lungs, kidneys, mesenteric lymph nodes, and spleen from one rat and the liver and mesenteric lymph nodes from another individual. *Yersinia* spp. were not isolated from *A. sylvaticus*.

PCR showed the presence of *ystB* in all *Y. enterocolitica* isolates. One isolate from *A. agrarius* had the two virulence markers, *ail* and *ystB*. The only isolate identified by API 20E as *Y. kristensenii* contained only the *ail* gene. The *ystA* gene was not found in any of the isolates.

*Yersinia* isolates confirmed by PCR were subjected to sero/biotyping. All obtained *Y. enterocolitica* isolates were salicyl-, eskulin-, and xylose-positive and since they exhibited the presence of pyrazinamidase activity and nitrate reduction, they were classified as BT 1A (Ye behavior). The *inv*-positive *Y. pseudotuberculosis* was classified as BT I (raffinose fermentation, melibiose fermentation and metabolic conversion of citrate) according to Niskanen et al. [24] and Tsubokura and Aleksic [4].

The results of the serotype affiliation are presented in Table 2. The majority of the 36 isolates did not react with any of the available diagnostic sera and were defined as NI (non-identified). 

The serotyping results confirmed the differentiation of double isolates from *A. agrarius* 203a and 203bApodemus agrarius (NI and O:3, respectively) and 209a and 209bApodemus agrarius (O:5 and NI, respectively). For isolates, 204a and bRattus norvegicus serotypes were not established (Table 2).

**Figure 1:**
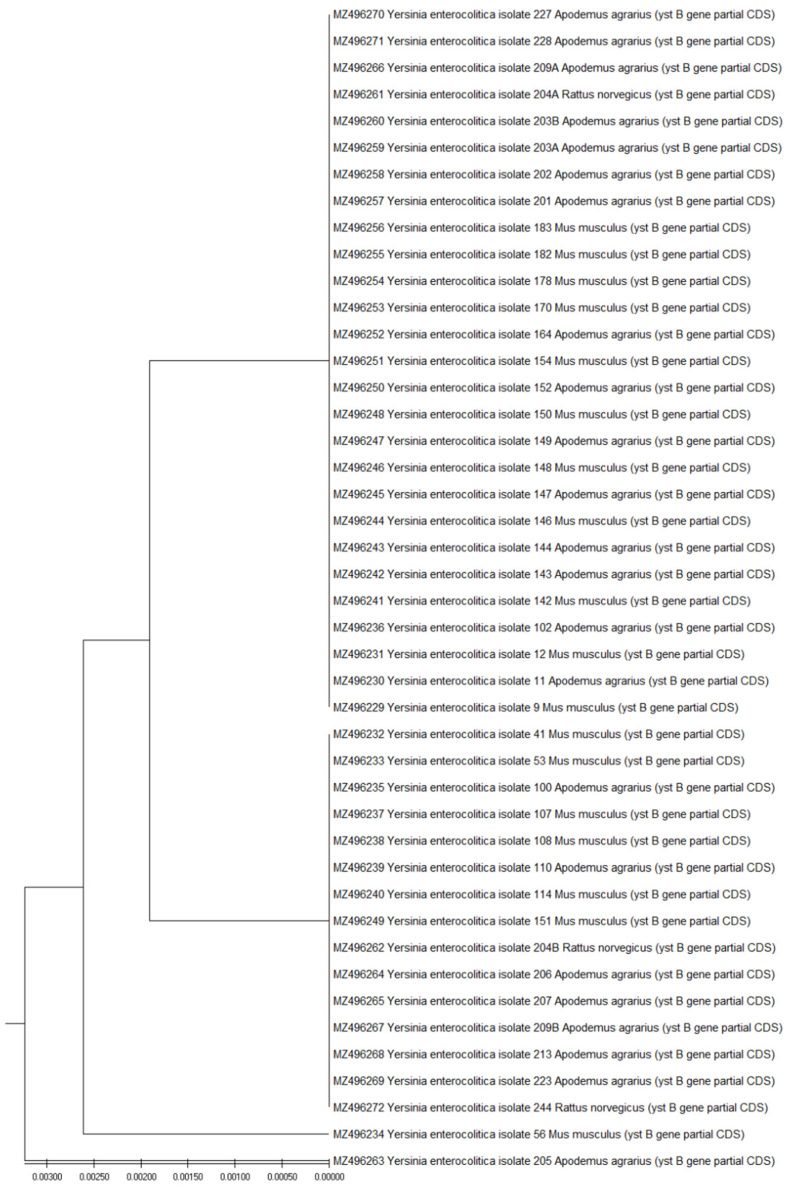
Evolutionary relationships of taxa.

Sequencing results confirmed the species affiliation of the isolates. Most of the sequenced *ystB* fragments were contained in two clusters. Only the *ystB* sequences of the 56Mus musculus and 205Apodemus agrarius isolates were located in separate branches (Figure 1).

The evolutionary history was inferred using the UPGMA method [28]. The optimal tree is shown. The tree is drawn to scale, with branch lengths in the same units as those of the evolutionary distances used to infer the phylogenetic tree. The evolutionary distances were computed using the Maximum Composite Likelihood method [29] and are in the units of the number of base substitutions per site. This analysis involved 44 nucleotide sequences. Codon positions included were 1st + 2nd + 3rd + Noncoding. All ambiguous positions were removed for each sequence pair (pairwise deletion option). There were a total of 264 positions in the final dataset. Evolutionary analyses were conducted in MEGA X [30].

The results of the sequencing of *ystB* fragments confirmed the differentiation of the double-isolated isolates from the field mice 209aApodemus agrarius and 209bApodemus agrarius, and 204aRattus Norvegicus and 204b from the rat. In both cases, sequences “a” and “b” were located in different clusters. The sequences of *ystB* fragments in both isolates from the field mouse 203aApodemus agrarius and 203b were the same, the difference was that the isolate 203aApodemus agrarius also contained the *ail* gene.

The partial coding sequence (cds) of *ystB* 263 base pairs (bp) long, from 11 *M. musculus* [MZ496229, MZ496231, MZ496241, MZ496244, MZ496246, MZ496248, MZ496251, MZ496253-MZ496256], 15 striped field mice [MZ496230, MZ496236, MZ496242, MZ496243, MZ496245, MZ496247, MZ496250, MZ496252, MZ496257-MZ496260, MZ496266, MZ496270, MZ496271] and from 1 *R. norvegicus* [MZ496261] were grouped in one cluster and all of these sequences demonstrated 100% similarity to the *ystB* sequences in *Y. enterocolitica* isolated from a beaver [Acc. No. KJ592623] in northern Poland, common voles [Acc. No. MK734430, MK734429.1] in southeastern Poland, from a food sample in South Korea [Acc No CP009456.1], and from a human host fecal sample in the UK [Acc. No. HF571988.1].

The second group of sequences was located in a separate cluster. The cds of *ystB* from six *M. musculus* [MZ496232, MZ496233, MZ496237, MZ496238, MZ496240, MZ496249], from seven *A. agrarius* [MZ496235, MZ496239, MZ496264, MZ496265, MZ496267-MZ496269], and from two *R. norvegicus* [MZ496262, MZ496272] demonstrated 100% similarity to the *ystB* sequences in *Y. enterocolitica* isolated from common voles [Acc. No. MK734428.1, MK734423.1, MK734422.1], yellow naked mice [MK734426.1, MK734425.1, MK734424.1, MK734422.1] in southwestern Poland and from a beaver in northern Poland [KJ592624.1]. 

The cds of the first of the separately located *ystB* fragments from the *M. musculus* isolate [MZ496234] had no Gene Bank counterpart with 100% similarity, but they demonstrated 99.625% similarity to the *ystB* sequences in *Y. enterocolitica* isolated from common voles [Acc. No. MK734430.1, MK734429.1] in southeastern Poland and a human host fecal sample in UK [HF571988.1] and 98.48% similarity to *ystB* from a mallard duck in northern Poland [KU198401.1]. The second different sequence from *A. agrarius* [MZ496263] showed 100% similarity to the *ystB* sequence in enterotoxin producing *Y. enterocolitica* 1A strain in India [Acc. No. AY966880.1], beaver [Acc. No. KJ592627.1] in Poland, and 99.62% similarity to sequences from common voles [Acc. No. MK734428.1, MK734423.1] and yellow naked mice [Acc. No. MK734424.1, MK734425.1, MK734427.1] from southeastern Poland.

Partial cds analysis of the *ail* gene (356 bp) from *Y. enterocolitica* isolated from *A. agrarius* showed 100% similarity to the sequences derived from *Y. enterocolitica* 1A isolated from a raw pork sample in Germany [Acc. No. FR847859.1], a clinical stool sample in Finland [Acc. No. FN812732.1], and a wild boar in Poland [Acc. No. KM253257.1].

A partial cds analysis of the *ail* gene from *Y. kristensenii* isolated from *M. musculus* showed 76.55% similarity to the sequence derived from *Y. enetrocolitica* isolated from rats in China [Acc. No. JX972144.1] and 74.72% similarity to the *ail* sequence from *Y. enterocolitica* isolated from raw pork in Germany [Acc. No. FR847859.1], *Y. enterocolitica* isolated from clinical stool sample in Finland [Acc. No. FN812732.1] and 74.44% similarity to a sequence from *Y. enterocolitica* isolated from fatal septicemia in the USA [Acc. No. CP009846.1].

The partial cds of *inv* (567 bp), [Acc. No. MZ491082] from *Y. pseudotuberculosis* isolate was 100% identical to corresponding sequences of *Y. pseudotuberculosis* BTI strain isolated from a clinical human sample in France [Acc. No. CP033713.1], to invasive strains from Finland [Acc. No.HE805230.1–HE805218.1] and to a corresponding sequence of *Y. pseudotuberculosis* isolated from a striped field mouse in southeastern Poland (Acc. No. MZ491083).

## 4. Discussion

This is the first study on the prevalence and genetic analysis of isolates of *Yersinia* spp. isolated from commensal rodents in Poland. The authors’ previous research concerned the occurrence of these pathogens among species of small wild rodents inhabiting forest and field environments, which usually hibernate in winter and rarely reach households or farm buildings. 

The presented study showed widespread *Y. enterocolitica* among rodents potentially inhabiting farm objects. The presented research did not cover the situation inside these facilities. The presence of *Yersinia* spp in 17.6% of samples of rodents included in the study proves their important role in the transmission of bacteria. Although the isolates of *Y. enterocolitica* obtained in the current study contained the *ystB* gene (characteristic of strains belonging to BT 1A and commonly considered non-pathogenic), based on the literature data rodents can also be infected with pathogenic strains. In a study by Backhans et al. [31] in which 190 colon samples were tested by PCR, *Y. enterocolitica* was isolated from 5% of mice caught in the vicinity of pig farms. The obtained isolates included, among others, those belonging to the 4/O:3 bioserotypes. Hayashidani et al. [17] and Oda et al. [32] isolated highly virulent O:8, BT 1B bioserotypes of *Y. enterocolitica* from field mice with the same pulsotypes as those isolated from pigs. 

In comparing the authors’ own results with results reported in other studies on yersinia prevalence in small rodents, similar results were obtained in Great Britain, Scandinavian countries, Germany and France, where mainly isolates belonging to BT 1A were found in domestic mice [19,33,34,35]. Oda et al. [32] obtained a percentage of positive *Y. enterocolitica* samples, similar to the current study, where out of 560 tested animals, 15.7% of wild rodents showed the presence of this microorganism. 

The current study showed that rodents may be carriers of more than one strain of *Y. enterocolitica*. In three cases, two isolates were obtained from one animal serotypically and genotypically different from each other. Particularly noteworthy is the case of isolating two *Y. enetreocolitica* BT 1A isolates from one *A. agrarius*. The first isolate contained the *ystB* virulence marker typical for this biotype (203a Apodemus agrarius), while the second isolate, (203b Apodemus agrarius), contained *ystB* with the same nucleotide sequences as the first isolate but additionally had the *ail* virulence marker. This is interesting because the *ail* gene, controlling the Ail protein, which plays an important role during attachment and invasion processes, usually occurs in tandem with the *ystA* gene and is typical for pathogenic *Y. enterocolitica* biotypes 2–5 [36]. The comparison of the *ail* sequence of the isolate in the current study with that available in the GeneBank database showed 100% similarity to the sequence detected in *Y. enterocolitica* isolated from a clinical stool sample from Finland [Acc. No. FN812732.1]. This may suggest potential pathogenic properties of *ail/ystB*-positive strains. Therefore, the question arises of what mechanisms of attaching additional genetic structures are responsible for the formation of such previously unknown *Y. enterocolitica* strains and, above all, how it affects the pathogenetic abilities of the bacterium. Undoubtedly, this is a problem that deserves more in-depth research. To the best of our knowledge this is the first case of the detection of such an atypical isolate from a rodent, which is simultaneously a carrier of the typical isolate *Y. enterocolitica* BT 1A. The authors previously discussed the occurrence of such rare *ail/ ystB*-positive isolates detected in game animals in Poland [37]. 

The isolate found in one *M. musculus*, identified by API20E with 89.2% probability as *Y. kristensenii*, also contained the *ail* gene, and the sequences of its fragment differed from the *ail* sequences available in GeneBank database. In the available literature, there was only one article describing the *ail* presence in *Y. kristensenii* [38]. However, the isolate mentioned above did not show the simultaneous presence of the *yst* gene, unlike those reported by Joutsen et al. [38]. According to the authors cited above, the identification of *Y. kristensenii* based on the API20E result is subject to a certain margin of error. The result of sequencing the *ail* fragment of the isolate in the current study confirmed that it belongs to the genus *Yersinia*, but the exact determination of the species requires more detailed analyses.

In the current study, the results of the sequencing of *ystB* fragments showed relatively little variability. Importantly, the comparison of the studied sequences with the data available in the NCBI database showed no similarities to the analogous *ystB* fragments derived from *Y. enterocolitica* isolated from farm animals. This may be due to both the lack of correlation and the lack of availability of similar sequences in the NCBI database. Additionally, this result supports extending the research in the future by comparing isolates from rodents with isolates obtained from farm animals from the areas where these studies were conducted. 

*Y. pseudotuberculosis*, the second yersinia co-responsible for yersiniosis, due to its history, may be associated with rodents, as it was previously thought to be the cause of the disease known as rodenciosis [39]. Fukushima et al. identified pest rodents as an animal factor associated with a high risk of *Y. pseudotuberculosis* on pig farms [40]. The results of the current study confirm that rodents can also be carriers of *Y. pseudotuberculosis*, which is responsible for the most severe cases of yersiniosis, but there are no records in the literature confirming that this yersinia was more often isolated from rodents than from other animal species. Contrary to expectations, in the current study it was successfully isolated from only one house mouse. The results of the experiments of other authors indicate difficulties in the culturing of this bacteria [33]. Thus, such a small number of isolations may be partially due to the limitations of laboratory methods. These assumptions are confirmed by the results of the authors’ previous studies and studies by other authors. In a previous study by the authors, only one isolate of *Y. pseudotuberculosis* was isolated in 214 wild forest rodents [22]. In a study by Backhans et al. [33], similar to the current results, out of 190 colon samples from rodents, *Y. pseudotuberculosis* was detected in only one animal. 

It is more difficult to lure rats to rodenticide bait stations routinely used in rodent control actions. Rat invasions periodically occur in livestock farms but not as frequently as mice invasions, which are seasonal in nature. Therefore, only two *R. norvegicus* were provided for the current study and they were caught in the areas of two different feed factories. It is noteworthy that the two rats that were lured into the traps were both infected with *Y. enterocolitica* and the bacteria was isolated from many of their internal organs. 

It is believed that because of the rodents that transmit the yersinia that it is impossible to obtain Yersinia-free herds in pig farms, despite following the strictest biosecurity rules [14,33]. The high prevalence percentage of yersinia in rodents in the current study may be related to the seasonality of rodent infestation in farm buildings occurring at the turn of autumn and winter. This coincides with a period of more frequent isolation of *Y. enterocolitica*, which has psychrophilic properties. However, none of the isolates obtained in the current study belonged to the so-called classic pathogenic biotypes that pose a threat to human health. 

## 5. Conclusions

The current study revealed a high prevalence of *Y. enetrocolitica* among commensal rodents. Although field mice were caught more frequently in the vicinity of farm buildings than domestic mice, *Y. enterocolitica* was more often isolated from intestinal samples of domestic mice. The classification of all obtained isolates into BT 1A of *Y. enterocolitica* and the sporadic isolation of *Y. pseudotuberculosis* do not indicate a high share of commensal rodents in the spreading of yersiniosis. However, the current study revealed that rodents may be carriers of more than one strain of *Y. enterocolitica,* including a new kind of BT 1A isolate with as yet unexplained pathogenic properties with the *ail* virulence marker.

## Figures and Tables

**Table 1:** Primer sequences for amplifying *ail, yst A, yst B,* and *inv* genes.

Gene	Primer Sequences	Product Size (bp—base pairs)	Source
*ail*	5′TGGTTATGCGCAAAGCCATGT3′ 5′TGGAAGTGGGTTGAATTGCA 3′	356	[25]
*ystA*	5′GTCTTCATTTGGAGGATTCGGC3′ 5′AATCACTACTGACTTCGGCTGG3′	134	[25]
*ystB*	5′TGTCAGCATTTATTCTCAACT3′ 5′GCCGATAATGTATCATCAAG3′	180	[26]
*inv*	CGGTACGGCTCAAGTTAATCTG CCGTTCTCCAATGTACGTATCC	183	[9]

**Table 2:** Characteristics of the *Yersinia* spp. isolates.

Isolate No.	Acc. No.	Yersinia	Gene	Biotype	Serotype	Source/Isolate Name
1.	MZ496229	*Y. enterocolitica*	*ystB*	1A	O:5	9 (Mus musculus)
2.	MZ496230	*Y. enterocolitica*	*ystB*	1A	O:5	11 (Apodemus agrarius)
3.	MZ496231	*Y. enterocolitica*	*ystB*	1A	NT	12 (Mus musculus)
4.	MZ496232	*Y. enterocolitica*	*ystB*	1A	NT	41 (Mus musculus)
5.	MZ496233	*Y. enterocolitica*	*ystB*	1A	NT	53 (Mus musculus)
6.	MZ491080	*Y. kristensenii*	*ail*		-	54 (Mus musculus)
7.	MZ496234	*Y. enterocolitica*	*ystB*	1A	NT	56 (Mus musculus)
8.	MZ491082	*Y. pseudotuberculosis*	*inv*	I	-	70 (Mus musculus)
9.	MZ496235	*Y. enterocolitica*	*ystB*	1A	NT	100 (Apodemus agrarius)
10.	MZ496236	*Y. enterocolitica*	*ystB*	1A	NT	102 (Apodemus agrarius)
11.	MZ496237	*Y. enterocolitica*	*ystB*	1A	NT	107 (Mus musculus)
12.	MZ496238	*Y. enterocolitica*	*ystB*	1A	NT	108 (Mus musculus)
13.	MZ496239	*Y. enterocolitica*	*ystB*	1A	NT	110 (Apodemus agrarius)
14.	MZ496240	*Y. enterocolitica*	*ystB*	1A	NT	114 (Mus musculus)
15.	MZ496241	*Y. enterocolitica*	*ystB*	1A	NT	142 (Mus musculus)
16.	MZ496242	*Y. enterocolitica*	*ystB*	1A	NT	143 (Apodemus agrarius)
17.	MZ496243	*Y. enterocolitica*	*ystB*	1A	NT	144 (Apodemus agrarius)
18.	MZ496244	*Y. enterocolitica*	*ystB*	1A	NT	146 (Mus musculus)
19.	MZ496245	*Y. enterocolitica*	*ystB*	1A	NT	147 (Apodemus agrarius)
20.	MZ496246	*Y. enterocolitica*	*ystB*	1A	NT	148 (Mus musculus)
21.	MZ496247	*Y. enterocolitica*	*ystB*	1A	NT	149 (Apodemus agrarius)
22.	MZ496248	*Y. enterocolitica*	*ystB*	1A	NT	150 (Mus musculus)
23.	MZ496249	*Y. enterocolitica*	*ystB*	1A	NT	151 (Mus musculus)
24.	MZ496250	*Y. enterocolitica*	*ystB*	1A	NT	152 (Apodemus agrarius)
25.	MZ496251	*Y. enterocolitica*	*ystB*	1A	NT	154 (Mus musculus)
26.	MZ496252	*Y. enterocolitica*	*ystB*	1A	O:5	164 (Apodemus agrarius)
27.	MZ496253	*Y. enterocolitica*	*ystB*	1A	NT	170 (Mus musculus)
28.	MZ496254	*Y. enterocolitica*	*ystB*	1A	NT	178 (Mus musculus)
29.	MZ496255	*Y. enterocolitica*	*ystB*	1A	NT	182 (Mus musculus)
30.	MZ496256	*Y. enterocolitica*	*ystB*	1A	O:5	183 (Mus musculus)
31.	MZ496257	*Y. enterocolitica*	*ystB*	1A	NT	201 (Apodemus agrarius)
32.	MZ496258	*Y. enterocolitica*	*ystB*	1A	NT	202 (Apodemus agrarius)
33.	MZ496259	*Y. enterocolitica*	*ystB*	1A	NT	203a (Apodemus agrarius)
34.	MZ496260 MZ491081	*Y. enterocolitica*	*ystB, ail*	1A	O:3	203b (Apodemus agrarius)
35.	MZ496261	*Y. enterocolitica*	*ystB*	1A	NT	204a (Rattus norvegicus)
36.	MZ496262	*Y. enterocolitica*	*ystB*	1A	NT	204b (Rattus norvegicus)
37.	MZ496263	*Y. enterocolitica*	*ystB*	1A	NT	205 (Apodemus agrarius)
38.	MZ496264	*Y. enterocolitica*	*ystB*	1A	NT	206 (Apodemus agrarius)
39.	MZ496265	*Y. enterocolitica*	*ystB*	1A	NT	207 (Apodemus agrarius)
40.	MZ496266	*Y. enterocolitica*	*ystB*	1A	O:5	209a (Apodemus agrarius)
41.	MZ496267	*Y. enterocolitica*	*ystB*	1A	NT	209b (Apodemus agrarius)
42.	MZ496268	*Y. enterocolitica*	*ystB*	1A	NT	213 (Apodemus agrarius)
43.	MZ496269	*Y. enterocolitica*	*ystB*	1A	NT	223 (Apodemus agrarius)
44.	MZ496270	*Y. enterocolitica*	*ystB*	1A	O:8	227 (Apodemus agrarius)
45.	MZ496271	*Y. enterocolitica*	*ystB*	1A	O:8	228 (Apodemus agrarius)
46.	MZ496272	*Y. enterocolitica*	*ystB*	1A	NT	244 (Rattus norvegicus)

## Data Availability

Not applicable.
